# Refractive outcomes after immediate primary phacoemulsification for acute primary angle closure

**DOI:** 10.1038/s41598-023-40585-9

**Published:** 2023-08-16

**Authors:** Takafumi Suzuki, Yoshiki Ueta, Naoko Tachi, Yasuhiro Okamoto, Takao Fukutome, Hirofumi Sasajima

**Affiliations:** 1 Department of Ophthalmology, Shinseikai Toyama Hospital, Imizu, Japan; 2 Department of Ophthalmology, The University of Tokyo Hospital, 7-3-1 Hongō, Bunkyō-ku, Tokyo, 113-8655 Japan; 3 Tachi Eye Clinic, Toyama, Japan; 4 Yamada Eye Clinic, Nagano, Japan

**Keywords:** Medical research, Eye diseases

## Abstract

This study investigated the refractive outcomes of 64 eyes overall including 32 immediate primary phacoemulsification in acute primary angle closure (APAC) eyes and 32 of their fellow eyes. We investigated best-corrected visual acuity, intraocular pressure (IOP), average keratometric diopter (K), spherical equivalent, axial length (AL), central corneal thickness, and anterior chamber depth (ACD) at preoperative examination (Pre) and more than 1-month post-phacoemulsification (1 m), and changes in values. Using SRK/T, Barrett Universal II (Barrett), Hill-Radial Basis Function Version 3.0 (RBF 3.0), and Kane formulas, we calculated and compared refractive prediction error (PE), absolute value of PE (AE), and changes in K, AL, and ACD from Pre to 1 m between APAC and fellow eyes. From Pre to 1 m, K remained similar in APAC and fellow eyes (p = 0.069 and p = 0.082); AL significantly decreased in APAC and in fellow eyes (both p < 0.001); and ACD significantly increased in APAC and in fellow eyes (both p < 0.001). The change in AL differed significantly between the two groups (p = 0.007). Compared to the fellow eyes, PE with SRK/T and Barret formulas (p = 0.0496 and p = 0.039) and AE with Barrett and RBF 3.0 formula (p = 0.001 and p = 0.024) were significantly larger in the APAC eyes. Thus, attention should be paid to refractive prediction error in immediate primary phacoemulsification for APAC eyes caused by preoperative AL elongation due to high IOP.

## Introduction

Acute primary angle closure (APAC) is an ocular disease requiring urgent treatment. Common treatments include medical therapy, such as miosis eye drops to remove the pupillary block, and intraocular pressure (IOP)-lowering eye drops, or intravenous infusion, followed by laser peripheral iridotomy (LPI)^1–5^ or phacoemulsification to remove the pupillary block^6–9^. We have previously reported the course of immediate phacoemulsification as a primary treatment for APAC with or without preoperative IOP-lowering medication (glaucoma eye drops, oral medications, or intravenous infusion)^10^.

IOL power calculation before cataract surgery for eyes with a short axial length (AL) is problematic in general^11–14^, and it can also be difficult in cases undergoing the surgery for APAC^15,16^. Refractive prediction error (PE) caused by corneal edema due to high IOP could be more problematic when phacoemulsification is performed very early, before IOP is sufficiently lowered preoperatively, than when it is performed after IOP is sufficiently lowered with medication or LPI.

To the best of our knowledge, no previous report on APAC eyes has examined the post-phacoemulsification refractive outcomes based on pre- and postoperative average keratometric diopter (K), AL, central corneal thickness (CCT), and anterior chamber depth (ACD), or has compared them to their fellow eyes by using these parameters. Therefore, in this study, we retrospectively investigated the refractive outcomes of immediate phacoemulsification as the primary treatment for APAC eyes and compared these with the refractive outcomes of their fellow eyes.

## Materials and methods

### Ethics

This study adhered to the tenets of the Declaration of Helsinki and was approved by the Institutional Review Board of Shinseikai Toyama Hospital (approval number: 230224-4). Our study was a retrospective consecutive series. We used an opt-out consent process, and the requirement for obtaining informed patient consent was waived by the institutional review board of Shinseikai Toyama Hospital.

### Patients

The patient database at the Shinseikai Toyama Hospital was searched, and the records of patients with APAC who visited our hospital between October 2009 and January 2023 were reviewed. We included patients with a history of intermittent blurring of vision, with halos and at least two of the following symptoms: ocular or periocular pain, and nausea and/or vomiting in APAC eyes. Additionally, for inclusion, patients had to have an IOP > 30 mmHg and the presence of at least three of the following signs: conjunctival injection, corneal epithelial edema, mid-dilated pupil, shallow anterior chamber, and iris atrophy in APAC eyes. Moreover, we included these patients if they underwent phacoemulsification in both the APAC eye and the fellow eye in our hospital and had more than 1 month of follow-up data available. Included patients had to have measurable preoperative K using any ophthalmokeratometer, optical AL, CCT, and ACD for both eyes.

We excluded patients with secondary glaucoma, such as neovascular glaucoma, phacolytic glaucoma, and uveitic glaucoma; patients with pseudophakic eyes, aphakic eyes, or eyes that underwent LPI; patients whose preoperative optical AL was not measurable due to severe corneal edema or whose signal-to-noise ratio was less than 2, and whose AL was measured with A-mode biometry using an ultrasound imager; as well as patients whose keratometric diopter was not measurable using our ophthalmokeratometer due to severe corneal edema.

### Ophthalmic examinations

All patients in this study underwent comprehensive ocular examinations, including best-corrected visual acuity (BCVA) measurement, IOP measurement with noncontact tonometry (Nidek NT-530, Gamagori, Japan), K measurement with an ophthalmokeratometer (Topcon KR-8100A, Tokyo, Japan or Nidek ARK-1, Gamagori, Japan), CCT measurement with a CASIA (Tomey, Nagoya, Japan), ACD measurement with a CASIA, AL measurement with an IOL Master 500 (Carl Zeiss Meditec, Germany) or OA2000 (Tomey, Nagoya, Japan), and slit-lamp biomicroscopy. The IOL power was calculated using the SRK/T, Barrett Universal II (Barrett), Hill-Radial Basis Function (RBF) 3.0, and Kane formula. We measured AL in the phakic mode preoperatively and in the pseudophakic mode postoperatively. Patients were followed up at more than 1 month after phacoemulsification.

### Treatment protocol

All patients underwent phacoemulsification within days after the onset of APAC. None of the included patients underwent LPI.

After the arrival of the patient at the hospital, we completed the ophthalmic examination and the tests necessary prior to surgery and for IOL power selection, initiated topical administration of tropicamide and phenylephrine, instead of LPI, and performed phacoemulsification as soon as possible. We administered IOP-lowering eye drops, acetazolamide, or glycerol systemically until the surgery if the patient experienced severe headache or nausea.

Phacoemulsification with IOL implantation was performed by two surgeons (NT and YU) under topical and sub-Tenon anesthesia. A 2.2-mm superior scleral or corneal incision was made. After phacoemulsification, a foldable 6.0-mm IOL (SN60WF; Alcon, Fort Worth, TX, USA or ZCB00V; AMO, Santa Ana, CA, USA) was implanted into the capsular bag. In addition, the anterior segment was refilled with viscoelastic and the angle was observed with a Mori goniotomy lens (RE Medical Inc., Osaka, Japan) after the insertion of the IOL. Goniosynechialysis (GSL) was performed without causing angle dissection if peripheral anterior synechiae (PAS) were visible. The surgeons placed a viscoelastic material near the PAS and gently pressed it against the peripheral iris with a blunt needle to exert backward pressure on the iris and to expose the trabecular meshwork. The same procedure was performed using a Nagata GSL needle (Inami, Japan) if the PAS persisted. GSL was stopped before goniodialysis occurred if the PAS persisted even after performing these techniques.

After the condition of the operated APAC eyes settled, the same surgery was performed on the fellow eyes to prevent APAC.

Postoperative treatment included administering a combination of moxifloxacin 0.5% three times daily, fluorometholone 0.1% or betamethasone 0.1% four times daily, and bromfenac 0.1% twice daily. This treatment was tapered over a period of a month. Pilocarpine 2% was given three times daily within a month from the surgery in some cases.

### Evaluation of the surgical outcomes and refractive outcomes of immediate phacoemulsification

After the preoperative examination (Pre) and surgery, all the patients were followed-up for more than 1 month (1 m).

For all patients, we recorded the time from APAC onset to the start of surgery for APAC; the time from arrival at the hospital to the start of the surgery; BCVA, IOP, K, spherical equivalent (SE), AL, CCT, and ACD at Pre and 1 m, and the changes in the values between these time-points; as well as surgery time.

We compared the PE [i.e., the postoperative actual refraction at 1 m minus the preoperative refraction predicted by the formula used to calculate the exact power of the implanted IOL (i.e., the Prediction)], absolute value of PE (AE), median absolute value of refractive error (MedAE), the Prediction, and the change in K, AL, and ACD from Pre to 1 m between the APAC eyes and the fellow eyes. We used the optimized constant of the User Group of the Laser Interference Biometry website for the SRK/T formula. The constants for the Barrett (available at https://calc.apacrs.org/barrett_universal2105/), RBF 3.0 (available at https://rbfcalculator.com/online/index.html), and Kane formulas (available at https://www.iolformula.com) were provided by an online calculator. We used the same optimized constant for both ZCB00V and ZCB00.

To investigate the effect of GSL on refractive outcome, we divided the APAC eyes and the fellow eyes into two groups, respectively. One group of eyes underwent intraoperative GSL, while the other were without intraoperative GSL. We compared PE and AE between the two groups of eyes in both the APAC and the fellow eye groups.

To evaluate the difference in accuracy of IOL power calculation for each formula, we compared PE and AE calculated using the four different formulas in each of the APAC eyes and the fellow eyes.

The main outcome of this study was to investigate the PE and AE in the APAC eyes as well as their causes using K, AL, and ACD, compared with those in the fellow eyes. The secondary outcome was to compare the accuracy of the IOL calculation formulas.

### Statistical analysis

Continuous variables are presented as mean (± standard deviation). BCVA was converted to the logarithm of the minimal angle of resolution.

The Wilcoxon signed-rank test was used to compare BCVA, IOP, K, SE, AL, CCT, and ACD values at Pre and at 1 m between APAC eyes and fellow eyes, as well as the change from Pre to 1 m within both the APAC and the fellow eye groups. This test was also used to compare PE, AE, and Prediction between APAC eyes and fellow eyes.

Mann–Whitney U test was used to compare PE and AE between eyes with and without GSL in both the APAC and fellow eye groups.

Kruskal–Wallis test was used to compare PE and AE between SRK/T, Barrett, RBF 3.0, and Kane formulas, in both the APAC and fellow eye groups.

Statistical significance was set at p < 0.05. Statistical analyses were performed using R version 4.0.2 (The R Foundation for Statistical Computing, Vienna, Austria).

### Institutional review board statement

The study was conducted in accordance with the Declaration of Helsinki and approved by the Institutional Review Board of Shinseikai Toyama Hospital (approval number 230224-4, February 24th, 2023).

### Informed consent statement

The need to obtain informed patient consent was waived by the institutional review board of Shinseikai Toyama Hospital due to the retrospective nature of the study and the use of an opt-out consent process.

## Results

### Patient characteristics

Five patients were excluded either because K measurement was not possible using any ophthalmokeratometer, optical AL measurement was not possible, or A-mode biometry with an ultrasound imager was used for AL measurement due to severe corneal edema. Three patients were excluded because they had previously undergone phacoemulsification of the fellow eye at another hospital and we had no preoperative data for the fellow eye. Another two patients were excluded because their fellow eye had chronic angle closure glaucoma, which was inappropriate as control for the APAC eye. One patient was excluded because he had anisotropic amblyopia and we considered that there was a difference in ocular morphology between his right and left eye. Thus, 64 eyes of 32 patients were finally included in this study.

The characteristics of the patients are presented in Table 1. The mean age of patients was 74.7 ± 8.1 years. The patients included six men (12 eyes) and 26 women (52 eyes). One case has pseudoexfoliation syndrome without lens dislocation in both eyes. This comorbidity was not considered to have significantly confounded the study results. All patients underwent phacoemulsification for APAC eyes within a day of their initial visit to our hospital. In the APAC eyes, the mean time from the initial visit to our hospital to phacoemulsification was 6.3 ± 7.7 h. The mean time from APAC onset to phacoemulsification was 50.6 ± 45.8 h (Table 1). In the fellow eyes, the phacoemulsification was performed within 1 month after phacoemulsification of the APAC eyes.

**Table 1:** Summary of results for the current study.

	APAC	Control	p value
Number of eyes	32	32	
Male	6	6	
Female	26	26	
Age (years)	74.7 ± 8.1		
Time from onset to operation (h)	50.6 ± 45.8		
Time from arrival to operation (h)	4.1 ± 5.8		
Operation time (min)	19.2 ± 8.2	13.4 ± 7.3	< 0.001*
BCVA (LogMAR)			
Pre	1.09 ± 0.86	0.10 ± 0.22	< 0.001*
1 m	0.14 ± 0.25^†^	− 0.001 ± 0.13^†^	0.001*
IOP (mmHg)			
Pre	45.0 ± 19.5	14.8 ± 5.0	0.002*
1 m	12.7 ± 2.2^†^	12.0 ± 2.5^†^	0.194
K (D)			
Pre	44.7 ± 1.5	44.6 ± 1.3	0.104
1 m	44.5 ± 1.5	44.3 ± 2.7	0.285
SE (D)			
Pre	0.32 ± 2.29	1.00 ± 1.98	0.018*
1 m	− 0.12 ± 0.58^††^	− 0.18 ± 0.58^†,†††^	0.518
AL (mm) (15 pairs of eyes)			
Pre	22.6 ± 0.5	22.4 ± 0.6	0.012*
1 m	22.3 ± 0.5^†^	22.3 ± 0.6^†^	0.252
CCT (μm) (16 pairs of eyes)			
Pre	594.4 ± 50.8	558.8 ± 45.3	0.002*
1 m	552.1 ± 41.4^†^	556.3 ± 48.6	0.204
ACD (μm) (16 pairs of eyes)			
Pre	1.52 ± 0.36	1.80 ± 0.29	0.005
1 m	3.72 ± 0.32^†^	3.82 ± 0.22^†^	0.423
Prediction (D) (SRK/T)	− 0.56 ± 0.37	− 0.41 ± 0.35	0.014*
Prediction (D) (Barrett)	− 0.68 ± 0.41	− 0.51 ± 0.40	0.055
Prediction (D) (RBF 3.0)	− 0.73 ± 0.40	− 0.57 ± 0.36	0.063
Prediction (D) (Kane)	− 0.57 ± 0.43	− 0.40 ± 0.36	0.059
PE (D) (SRK/T)	0.45 ± 0.62	0.23 ± 0.61	0.0496*
PE (D) (Barrett)	0.57 ± 0.68	0.33 ± 0.60	0.039*
PE (D) (RBF 3.0)	0.61 ± 0.63	0.39 ± 0.57	0.070
PE (D) (Kane)	0.45 ± 0.68	0.22 ± 0.59	0.077
AE (D) (SRK/T)	0.67 ± 0.38	0.49 ± 0.40	0.102
AE (D) (Barrett)	0.77 ± 0.44	0.54 ± 0.41	0.001*
AE (D) (RBF 3.0)	0.77 ± 0.41	0.54 ± 0.41	0.024*
AE (D) (Kane)	0.70 ± 0.41	0.50 ± 0.36	0.111
MedAE (D) (SRK/T)	0.57	0.36	–
MedAE (D) (Barrett)	0.77	0.45	–
MedAE (D) (RBF 3.0)	0.76	0.38	–
MedAE (D) (Kane)	0.62	0.40	–

*Significantly different between the two group (p < 0.05).

^†^Significantly different from the preoperative period (p < 0.05).

^††^Significantly different from the Prediction value calculated by the SRK/T, Barrett, RBF 3.0, and Kane formulas (p < 0.05).

^†††^Significantly different from the Prediction value calculated by the Barret, RBF 3.0, and Kane formulas (p < 0.05).

*APAC* group of eyes with acute primary angle closure, *Control* group of fellow eyes, *h* hour, *min* minute, *BCVA* best corrected visual acuity, *LogMAR* Converted to the logarithm of the minimal angle of resolution, *IOP* intraocular pressure, *K* keratometric diopter, *SE* spherical equivalent, *AL* axial length, *CCT* central corneal thickness, *ACD* anterior chamber depth, *Prediction* predicted SE, *Barrett* Barrett Universal II, *RBF 3.0* Hill-Radial Basis Function Calculator Version 3.0, *PE* mean refractive prediction error, *AE* mean absolute refractive prediction error, *MedAE* median absolute refractive prediction error, *Pre* preoperative period, *1 m* more than 1 month after phacoemulsification.

### Operation and complications

In the 32 APAC eyes, the operative time was 19.2 ± 8.2 min (Table 1). Three eyes required corneal epithelial abrasion because of intraocular transparency. The surgeons covered the eyes with therapeutic contact lens at the end of surgery. The lens were removed within 1 week after surgery, and corneal epithelial defects healed within 2 weeks after surgery in all the three eyes. GSL was performed in 19 eyes. The IOL was inserted into the bag in all cases, and no severe adverse events were observed. Small surgical complications included iris prolapse in two eyes and anterior chamber hemorrhage in one eye. On postoperative day 1, nine eyes had anterior chamber fibrin, and three eyes had anterior chamber hemorrhage, which improved within a few days. None of the patients required reoperation within the follow-up period.

In the 32 fellow eyes, the operative time was 13.4 ± 7.3 min (Table 1). GSL was performed in 10 eyes. The IOL was inserted into the bag in all fellow eyes, and no severe adverse events were observed. Small surgical complications included iris prolapse in one eye and main wound burn in one eye, which required suturing. On postoperative day 1, two eyes demonstrated anterior chamber fibrin, which improved within a few days. None of the fellow-eyes required reoperation within the follow-up periods.

There was a significant difference in the operative time between the APAC eyes and their fellow eyes (p = 0.001) (Table 1).

### Comparison of changes in BCVA, IOP, K, SE, AL, CCT, and ACD from Pre to 1 m, and calculation of Prediction, PE, and AE each in APAC eyes and in their fellow eyes

Values of all parameters of interest in the APAC eyes and fellow eyes are listed in Table 1. The AL, CCT, and ACD of 15 or 16 pairs of eyes at both Pre and 1 m were measured and compared due to the lack of postoperative AL, CCT, and ACD data, because of the retrospective study design. For APAC eyes, BCVA was significantly improved at 1 m as compared to that at Pre (p < 0.001). IOP was significantly decreased at 1 m as compared to that at Pre (p < 0.001). K and SE remained unchanged from Pre to 1 m (p = 0.069 and p = 0.090, respectively). AL and CCT significantly decreased from Pre to 1 m (both p < 0.001), whereas ACD significantly increased from Pre to 1 m (p < 0.001). Prediction values calculated by SRK/T, Barret, RBF 3.0, and Kane formulas were − 0.56 ± 0.37 (D), − 0.68 ± 0.41 (D), − 0.73 ± 0.40 (D), and − 0.57 ± 0.43 (D), respectively, while PE values calculated by these formulas were 0.45 ± 0.62 (D) , 0.57 ± 0.68 (D), 0.61 ± 0.63 (D), and 0.45 ± 0.68 (D) respectively. SE at 1 m was significantly different from the Prediction values calculated by the SRK/T, Barret, RBF 3.0, and Kane formulas (all p < 0.001). AE values calculated by these formulas were 0.67 ± 0.38 (D), 0.77 ± 0.44 (D), 0.77 ± 0.41(D), and 0.70 ± 0.41 (D), respectively, while the MedAE values were 0.57 (D), 0.77 (D), 0.76 (D), and 0.62 (D), respectively (Table 1).

For the fellow eyes, BCVA was significantly improved at 1 m as compared with that at Pre (p < 0.001). IOP was significantly decreased at 1 m as compared with that at Pre (p < 0.001). K was not significantly different from Pre to 1 m (p = 0.082), but SE changed significantly from Pre to 1 m in the fellow eyes (p = 0.002). AL and CCT significantly decreased from Pre to 1 m (p < 0.001 and p = 0.002, respectively). ACD increased significantly from Pre to 1 m (p < 0.001). Prediction values calculated by SRK/T, Barret, RBF 3.0, and Kane formulas were − 0.41 ± 0.35 (D), − 0.51 ± 0.40 (D), − 0.57 ± 0.36 (D), and − 0.40 ± 0.36 (D), respectively. PE values calculated by these formulas were 0.23 ± 0.61 (D), 0.33 ± 0.60 (D), 0.39 ± 0.57 (D), and 0.22 ± 0.59 (D), respectively. SE at 1 m was significantly different from the Prediction value calculated with the Barret, RBF 3.0, and Kane but not from that calculated with the SRK/T formula (p = 0.008, p = 0.001, p = 0.0497, and p = 0.063, respectively. AE values calculated by these formulas were 0.49 ± 0.40 (D), 0.54 ± 0.41 (D), 0.54 ± 0.41(D), and 0.50 ± 0.36 (D), respectively, while the MedAE values were 0.36 (D), 0.45 (D), 0.38 (D), and 0.40 (D), respectively (Table 1).

### Comparison of BCVA, IOP, K, SE, AL, CCT, and ACD at Pre and at 1 m, and of prediction, PE, and AE between APAC eyes and their fellow eyes

BCVA in APAC eyes was significantly worse than that in the fellow eyes at Pre and at 1 m (p < 0.001 and p = 0.001, respectively). The IOP in APAC eyes was significantly higher than that in the fellow eyes at Pre, but not at 1 m (p < 0.001 and p = 0.194). K in APAC eyes was not significantly different from that in the fellow eyes at Pre or at 1 m (p = 0.104 and p = 0.285, respectively). SE in APAC eyes was significantly different from that in the fellow eyes at Pre, but not at 1 m (p = 0.018 and p = 0.518, respectively). AL in APAC eyes was significantly longer than that in the fellow eyes at Pre, but not at 1 m (p = 0.012 and p = 0.252, respectively). CCT in APAC eyes was significantly thicker than that in the fellow eyes at Pre, but not at 1 m (p < 0.001 and p = 0.204, respectively). The ACD in APAC eyes was significantly shallower than that in the fellow eyes at Pre, but not at 1 m (p = 0.005 and p = 0.423, respectively). The Prediction value calculated using the SRK/T formula was significantly different, but were not significantly different using the Barret, RBF 3.0, and Kane formulas between APAC and fellow eyes (p = 0.014, p = 0.055, p = 0.063, and 0.059, respectively). PE values calculated with the SRK/T and Barret formulas were significantly larger in the APAC eyes than those in the fellow eyes but were not significantly different using the RBF 3.0 and Kane formulas (p = 0.0496, p = 0.039, p = 0.070, and p = 0.077, respectively). AE values calculated by the Barret and RBF 3.0 formulas were significantly larger in the APAC eyes than those in the fellow eyes but were not significantly different using the SRK/T and Kane formulas (p = 0.001, p = 0.024, p = 0.102, and p = 0.111, respectively) (Table 1).

### Comparison of PE and AE using each formula in the APAC eyes and fellow eyes

**Figure 1:**
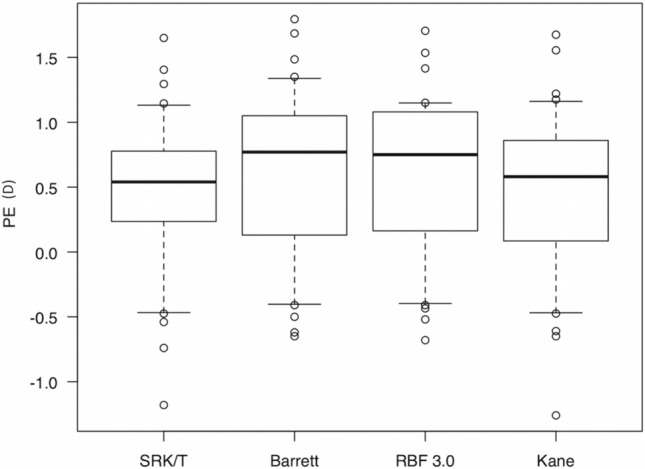
Comparison of PE of each formula in the APAC eyes. PE values with the SRK/T, Barrett, RBF 3.0, and Kane formulas were not significantly different in the APAC eyes (p = 0.432). *PE* mean refractive prediction error, *D* diopter, *Barrett* Barrett Universal II, *RBF 3.0* Hill-Radial Basis Function Calculator Version 3.0

**Figure 2:**
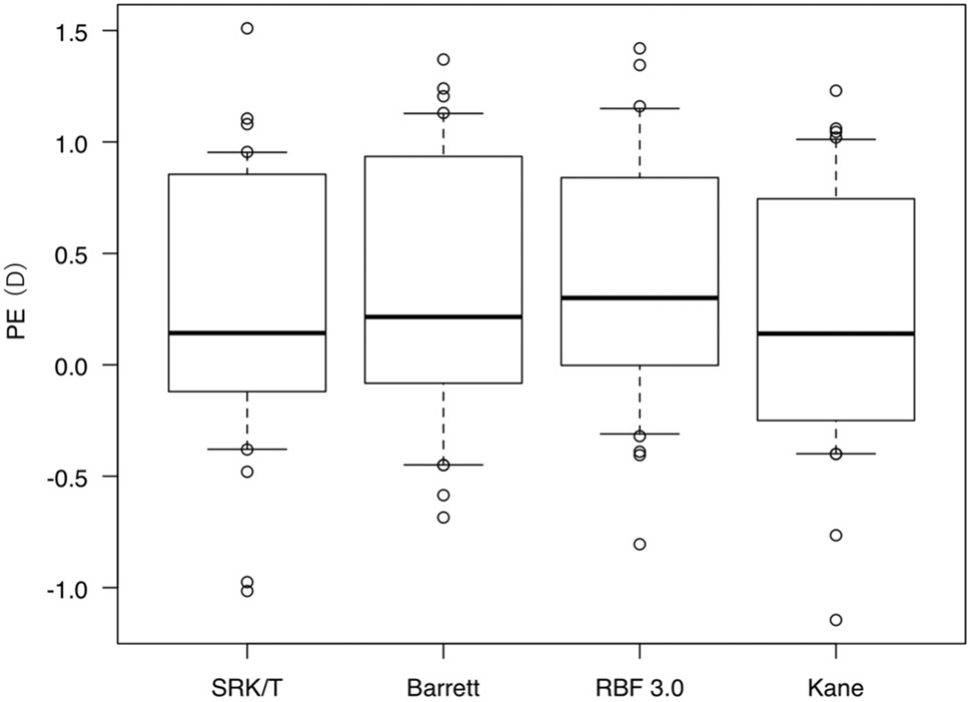
Comparison of PE of each formula in the fellow eyes. PE values with the SRK/T, Barrett, RBF 3.0, and Kane formulas were not significantly different in the fellow eyes (p = 0.599). *PE* Mean refractive prediction error, *D* diopter, *Barrett* Barrett Universal II, *RBF 3.0* Hill-Radial Basis Function Calculator Version 3.0

**Figure 3:**
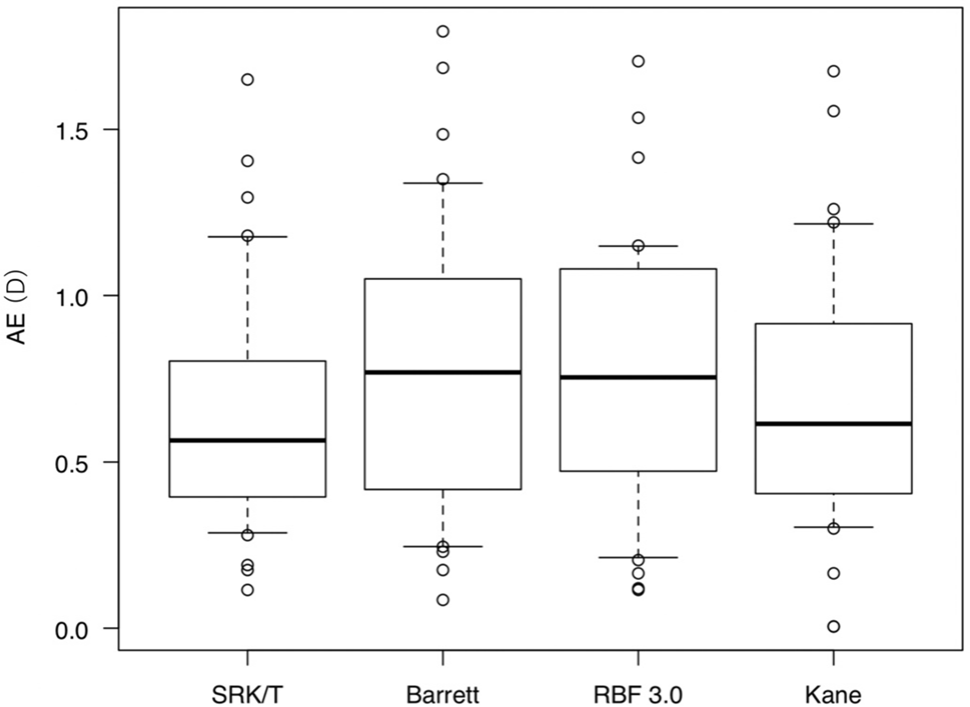
Comparison of AE of each formula in the APAC eyes. AE values with the SRK/T, Barrett, RBF 3.0, and Kane formulas were not significantly different in the APAC eyes (p = 0.546). *AE* Mean absolute refractive prediction error, *D* diopter, *Barrett* Barrett Universal II, *RBF 3.0* Hill-Radial Basis Function Calculator Version 3.0

**Figure 4:**
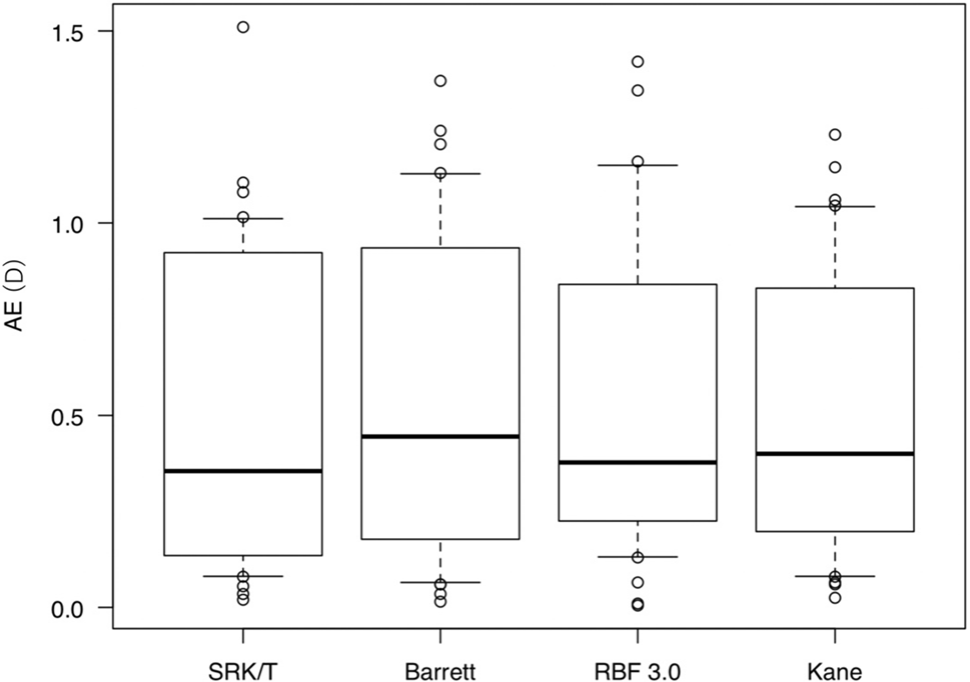
Comparison of AE of each formula in the fellow eyes. AE values with the SRK/T, Barrett, RBF 3.0, and Kane formulas were not significantly different in the fellow eyes (p = 0.897). *AE* Mean absolute refractive prediction error, *D* diopter, *Barrett* Barrett Universal II, *RBF 3.0* Hill-Radial Basis Function Calculator Version 3.0

Both in the APAC eyes and fellow eyes, PE values with the SRK/T, Barrett, RBF 3.0, and Kane formulas were not significantly different (p = 0.432 and p = 0.599) (Figs. 1, 2); similarly, those for AE values were also not significantly different (p = 0.546 and p = 0.897) (Figs. 3, 4).

### Comparison of PE and AE with or without GSL in the APAC eyes and fellow eyes

For the APAC eyes, PE and AE values were not significantly different with the SRK/T, Barrett, RBF 3.0, and Kane formulas between the eyes with and those without GSL (p = 0.291, p = 0.478, p = 0.545, and p = 0.514); (p = 0.673, p = 0.388, p = 0.362, and p = 0.759), respectively) (Table 2).

**Table 2:** Comparison of PE and AE with or without GSL in the APAC eyes.

Formula		GSL (+)	GSL (−)	p value
SRK/T	PE (D)	0.38 ± 0.69	0.57 ± 0.43	0.291
	AE (D)	0.67 ± 0.41	0.65 ± 0.29	0.673
	MedAE (D)	0.52	0.63	–
Barrett	PE (D)	0.52 ± 0.70	0.66 ± 0.60	0.478
	AE (D)	0.73 ± 0.47	0.81 ± 0.37	0.388
	MedAE (D)	0.62	0.84	–
RBF 3.0	PE (D)	0.60 ± 0.61	0.66 ± 0.63	0.545
	AE (D)	0.75 ± 0.42	0.83 ± 0.37	0.362
	MedAE (D)	0.64	0.98	–
Kane	PE (D)	0.42 ± 0.72	0.52 ± 0.54	0.514
	AE (D)	0.71 ± 0.44	0.68 ± 0.32	0.759
	MedAE (D)	0.60	0.75	–

GSL was performed in 19 eyes, but not in 13 eyes. There was no significant difference in PE or AE for all the four formulas between eyes with and without GSL.

*PE* mean refractive prediction error, *AE* mean absolute refractive prediction error, *GSL* goniosynechialysis, *APAC* acute primary angle closure, *D* diopter, *MedAE* median absolute refractive prediction error, *Barrett* Barrett Universal II, *RBF 3.0* Hill-Radial Basis Function Calculator Version 3.0

Similarly, for the fellow eyes, PE and AE values were not significantly different with the SRK/T, Barrett, RBF 3.0, and Kane formulas between the eyes with and those without GSL (p = 0.889, p = 0.675, p = 0.792, and p = 0.734); (p = 0.760, p = 0.675, p = 0.281, and p = 0.684), respectively) (Table 3).

**Table 3:** Comparison of PE and AE with or without GSL in the fellow eyes.

Formula		GSL (+)	GSL (−)	p value
SRK/T	PE (D)	0.23 ± 0.65	0.23 ± 0.57	0.889
	AE (D)	0.52 ± 0.45	0.48 ± 0.38	0.760
	MedAE (D)	0.36	0.36	–
Barrett	PE (D)	0.38 ± 0.63	0.30 ± 0.57	0.675
	AE (D)	0.61 ± 0.41	0.50 ± 0.41	0.489
	MedAE (D)	0.51	0.41	–
RBF 3.0	PE (D)	0.39 ± 0.63	0.39 ± 0.53	0.792
	AE (D)	0.62 ± 0.39	0.51 ± 0.42	0.281
	MedAE (D)	0.47	0.35	–
Kane	PE (D)	0.22 ± 0.62	0.22 ± 0.56	0.734
	AE (D)	0.53 ± 0.39	0.49 ± 0.34	0.684
	MedAE (D)	0.40	0.40	–

GSL was performed in 10 eyes, but not in 22 eyes. There was no significant difference in PE or AE for all the four formulas between eyes with and without GSL.

*PE* Mean refractive prediction error, *AE* mean absolute refractive prediction error, *GSL* goniosynechialysis, *D* diopter, *MedAE* median absolute refractive prediction error, *Barrett* Barrett Universal II, *RBF 3.0* Hill-Radial Basis Function Calculator Version 3.0

### Comparison of change in K, AL, and ACD from Pre to 1 m between APAC and fellow eyes

The change in K and ACD over the follow-up time was not significantly different between the two groups (p = 0.152 and p = 0.159, respectively). The change in AL was significantly larger in the APAC eyes than that in the fellow eyes (p = 0.007) (Table 4).

**Table 4:** Comparison of change in K, AL, and ACD between APAC and control.

	APAC	Control	p value
K change (D)	− 0.22 ± 0.60	− 0.12 ± 0.51	0.152
AL change (mm)	− 0.30 ± 0.17	− 0.12 ± 0.07	0.007*
ACD change (mm)	2.18 ± 0.42	2.11 ± 0.53	0.159

*APAC* Group of eyes with acute primary angle closure, *Control* group of fellow eyes, *K* keratometric diopter, *AL* axial length, *ACD* anterior chamber depth, *K change* Change of K from first visit to more than 1 month after surgery, *D* diopter, *AL change* Change of AL from first visit to more than 1 month after surgery, *ACD change* Change of ACD from first visit to more than 1 month after surgery.

## Discussion

To the best of our knowledge, there is no report that investigated the prediction error of IOL selection in phacoemulsification for APAC eyes with K, ACD, and AL, and compared these parameters to those of the fellow eyes. In our study, we showed that primary immediate phacoemulsification of APAC eyes resulted in greater refractive error in the far direction than that for the fellow eyes using the SRK/T and Barrett formulas, due to the difference in AL change. The RBF 3.0 and Kane formulas were not significantly different. Though, they also demonstrated similar tendencies for refractive error in the far direction for APAC eyes compared with fellow eyes as the SRK/T and Barrett formulas. Furthermore, AE values calculated with the SRK/T and Kane formulas in APAC eyes were not significantly different from those in the fellow eyes; nonetheless, with the Barrett and RBF 3.0 those in APAC eyes were significantly greater. There was no significant difference for PE or AE both in APAC eyes and the fellow eyes using all the four formulas including the SRK/T, Barrett, RBF 3.0, and Kane formulas. GSL using a viscoelastic material or needle did not significantly influence the refractive outcome in both APAC and fellow eyes.

Previous reports regarding the relationship between IOP and axial myopia have shown that IOP is positively correlated with high myopia^17^ as well as with axial myopia^18^. Leydolt et al.^19^ evaluated ocular biometric changes as a reaction to IOP changes in human eyes, and observed AL elongation when the IOP was increased by mechanical pressure on adult eyes. Another report^20^ showed that AL was shorter after than before phacoemulsification, although the mechanism is controversial.

As we previously reported^10^, primary phacoemulsification provides good postoperative IOP control. However, preoperative examination for selecting the appropriate IOL was performed very early, when IOP was still high, and AL was measured while still elongated. This was consistent with the finding that SE at Pre was significantly smaller in APAC eyes than that in the fellow eyes, which may be due to AL elongation. Thus, the postoperative refractive outcome shifted further distally in the APAC eyes than in the fellow eyes due to postoperative AL shortening.

For postoperative refractive error after phacoemulsification in patients who did not have APAC but had short AL, distal refractive error is problematic^11–14^. Thus, the postoperative refractive outcome was also considered to be shifted further distally in both APAC and control eyes in our study.

A previous study reported on refractive error after phacoemulsification for APAC eyes^15^. That study was a retrospective comparison of 36 APAC eyes before and at 6 months after surgery, without comparison of the fellow eye. They concluded that no significant difference was observed between the average preoperative Prediction value and the average postoperative SE. Although IOP at the initial visit was around 50 mmHg, they performed preoperative examination for IOL power selection in the presence of corneal edema, after patients had received medical therapy for ocular hypertension. The study provided no detailed description of preoperative IOP-lowering medication. Their time from APAC onset to surgery was about 8 days, which was longer than that in our study. They indicated that they had lowered IOP sufficiently with medication before performing the preoperative examination for IOL power selection. Therefore, we assumed that neither K nor AL were significantly changed and that there was no significant difference between the preoperative Prediction and postoperative SE values.

Another study reported on refractive error after phacoemulsification for primary angle closure glaucoma (PACG) eyes^16^. That retrospective study compared the refractive outcomes of 63 eyes with PACG with the results of 93 eyes with normal open angles undergoing uneventful phacoemulsification. They did not measure postoperative K, AL, or ACD. They showed that, although IOL power prediction may be inaccurate in PACG patients, there was no significant difference in PE between APAC eyes and control eyes. The preoperative average IOPs in the PACG and in the control group were normal. Thus, PE caused by AL elongation due to high IOP was not considered to be a problem.

A previous study reported the accuracy of IOL power calculation in primary angle‐closure disease (PACD) using seven formulas^21^ and showed a high performance of the new artificial-intelligence-based formulas including the Kane and RBF 3.0 formulas, as well as the traditional formula (SRK/T). In addition, they showed that the Kane formula achieved the best accuracy, while the SRK/T and Barrett formulas achieved satisfying performances when dealing with PACD eyes shorter than 22 mm. Our study showed that the SRK/T and Kane formulas may be less susceptible to acute parameter changes due to APAC than the Barrett and RBF 3.0 formulas with respect to AE when comparing them with the fellow eyes, which are morphologically similar to the PACD eyes in previous report. Considering the results of both previous and our study reports, the SRK/T and Kane formulas perform well in the PACD eyes including APAC eyes. However, the mean ACD in the previous report was 2.2 mm, which is deeper than that in our study. In addition, although preoperative IOP, corneal thickness, and corneal edema were not mentioned in the previous report, more than half of the eyes received peripheral iridectomy or trabeculectomy. They suggest that the previous study was performed on patients with lower preoperative IOP than that in our patients. Therefore, we cannot precisely compare the accuracy of the formulas between the previous study and ours.

The present study had several limitations. It was a retrospective, observational, small case-series study. Preoperative examination for IOL selection might be sufficiently unreliable due to corneal edema. In fact, some cases in which optical AL and K could not be measured reliably due to corneal edema were excluded. Using two types of IOL could have influenced the refractive outcomes, although in all pairs of eyes, the same type of IOL was inserted. Additionally, two devices were used for AL measurement over our long study period: the IOL Master 500 and the OA2000. However, the biometric parameters measured by these two biometers have shown good agreement^22^. Further studies are needed to examine postoperative PE and AE in a larger number of APAC patients.

## Conclusion

The results of our study indicate the need for attention to refractive prediction error when performing immediate primary phacoemulsification for APAC eyes caused by preoperative AL elongation due to high IOP.

## Data Availability

The data presented in this study are available upon request from the corresponding author.
